# Involving Older Adults During COVID-19 Restrictions in Developing an Ecosystem Supporting Active Aging: Overview of Alternative Elicitation Methods and Common Requirements From Five European Countries

**DOI:** 10.3389/fpsyg.2022.818706

**Published:** 2022-02-28

**Authors:** Kerli Mooses, Mariana Camacho, Filippo Cavallo, Michael David Burnard, Carina Dantas, Grazia D’Onofrio, Adriano Fernandes, Laura Fiorini, Ana Gama, Ana Perandrés Gómez, Lucia Gonzalez, Diana Guardado, Tahira Iqbal, María Sanchez Melero, Francisco José Melero Muñoz, Francisco Javier Moreno Muro, Femke Nijboer, Sofia Ortet, Erika Rovini, Lara Toccafondi, Sefora Tunc, Kuldar Taveter

**Affiliations:** ^1^Institute of Computer Science, Faculty of Science and Technology, University of Tartu, Tartu, Estonia; ^2^Department of Innovation, Santa Casa da Misericórdia da Amadora (SCMA), Amadora, Portugal; ^3^Department of Industrial Engineering, University of Florence, Florence, Italy; ^4^InnoRenew CoE, Izola, Slovenia; ^5^Andrej Marušič Institute, University of Primorska, Koper, Slovenia; ^6^Cáritas Diocesana de Coimbra (CDC), Coimbra, Portugal; ^7^Complex Unit of Geriatrics, Department of Medical Sciences, Fondazione “Casa Sollievo della Sofferenza”—IRCCS, Foggia, Italy; ^8^Fundación Ageing Lab, Mengibar, Spain; ^9^Technical Research Centre of Furniture and Wood of the Region of Murcia, Yecla, Spain; ^10^Telecommunication Networks Engineering Group, Technical University of Cartagena, Cartagena, Spain; ^11^Biomedical Signals and Systems, University of Twente, Enschede, Netherlands; ^12^Umana Persone, Grosseto, Italy

**Keywords:** requirements elicitation, elicitation methods, active aging, ecosystem, Pharaon, emotional goals, functional goals, quality goals

## Abstract

**Background:**

Information and communication technology solutions have the potential to support active and healthy aging and improve monitoring and treatment outcomes. To make such solutions acceptable, all stakeholders must be involved in the requirements elicitation process. Due to the COVID-19 situation, alternative approaches to commonly used face-to-face methods must often be used. One aim of the current article is to share a unique experience from the Pharaon project where due to the COVID-19 outbreak alternative elicitation methods were used. In addition, an overview of common functional, quality, and emotional goals identified by six pilot sites is presented to complement the knowledge about the needs of older adults.

**Methods:**

Originally planned face-to-face co-creation seminars were impossible to carry out, and all pilot sites chose alternative requirements elicitation methods that were most suitable in their situation. The elicited requirements were presented in the form of goal models. In one summary goal model, we provide an overview of common functional, quality, and emotional goals.

**Results:**

Different elicitation methods were combined based on the digital literacy of the target group and their access to digital tools. Methods applied without digital technologies were phone interviews, reviews of literature and previous projects, while by means of digital technologies online interviews, online questionnaires, and (semi-)virtual co-creation seminars were conducted. The combination of the methods allowed to involve all planned stakeholders. Virtual and semi-virtual co-creation seminars created collaborative environment comparable to face-to-face situations, while online participation helped to save the time of the participants. The most prevalent functional goals elicited were “Monitor health,” “Receive advice,” “Receive information.” “Easy to use/comfortable,” “personalized/tailored,” “automatic/smart” were identified as most prevalent quality goals. Most frequently occurring emotional goals were “involved,” “empowered,” and “informed.”

**Conclusion:**

There are alternative methods to face-to-face co-creation seminars, which effectively involve older adults and other stakeholders in the requirements elicitation process. Despite the used elicitation method, the requirements can be easily transformed into goal models to present the results in a uniform way. The common requirements across different pilots provided a strong foundation for representing detailed requirements and input for further software development processes.

## Introduction

Demographic change—the aging of the population—is a fortunate consequence of the positive evolution of care and wellbeing, but also presents big challenges to developed countries. To reduce the financial burden on the health sector, it is necessary to focus on improving the quality of life of older adults through supporting their independence and overall health. Advanced information and communication technology (ICT) solutions have the potential to address these challenges and support “aging in place,” meaning that older adults can remain healthy in their familiar home environments and retain independence for longer periods. Aging in place can be supported by several monitoring devices and smart home solutions, which allow to monitor the health status of older adults and provide guidance in the home setting (Demiris and Hensel, 2008; Moraitou et al., 2017; Vollenbroek-Hutten et al., 2017). Such solutions make both the older adult and the caregiver aware of the health situation and enable them to take action if necessary. Different ICT-based solutions of home care can provide assistance in daily activities, monitor the safety and security of home environments (Demiris and Hensel, 2008; Moraitou et al., 2017), provide access to cognitive or physical exercises which can be supervised remotely by professionals (Vollenbroek-Hutten et al., 2017), and support social interaction with family members and caregivers (Demiris and Hensel, 2008; Moraitou et al., 2017).

Incorporating ICT-based solutions that monitor the environment of an older adult and support her or his daily activities has several benefits. Besides the advantage of being able to stay at home, which is often more convenient for the older adult, the application of such solutions helps to reduce the burden of the healthcare and social care sectors (Polisena et al., 2009; Ekeland et al., 2010; Tappenden et al., 2012). In addition, the usage of ICT-based solutions in home care can improve the access to care and its outcomes (Lindberg et al., 2013), resulting in actual health behavior change, with an overall positive impact on life quality (Hirvonen et al., 2020). Moreover, the supportive effect of social connectedness has been pointed out (Lindberg et al., 2013; Zhang et al., 2020). Supporting social interactions of older adults is extremely important for their mental and physical health as it has been indicated that social interactions have the potential to support the healing process (Martino et al., 2017), have significant impact on mood and psychological health (Martino et al., 2017; Santini et al., 2020), and reduce mortality risk (Holt-Lunstad et al., 2010). Supporting social interactions of older adults has assumed even greater importance in the current COVID-19 situation where social distancing has become a norm and older adults, as the main risk group, are kept isolated. In such situations, ICT-based solutions become nearly irreplaceable in reducing the social isolation of older adults and retaining their supportive social networks. According to the previous studies, it is not only older adults who can benefit from ICT-based solutions while receiving home care, but also caregivers, who have reported that ICT-based solutions help to reduce their burden, improve overall mental health, and increase confidence in caregiving skills and competence (Godwin et al., 2013).

Therefore, advanced ICT-based solutions aimed at supporting aging in place offer multiple solutions for different stakeholders, which help to improve the quality of life. It is widely accepted that the approach of sociotechnical systems, which comprise human, social, organizational, and technical factors, leads to systems that are more acceptable to end users and delivers better value to stakeholders (Carayon, 2006; Sterling and Taveter, 2009; Baxter and Sommerville, 2011; Winby and Mohrman, 2018). The first step in developing sociotechnical ICT-based solutions supporting healthy and active aging should be to identify the problems faced by the stakeholders. More precisely, the needs, problems, capabilities, preferences, and characteristics of all the stakeholders should be uncovered and considered in the requirements engineering phase to ensure the usability and acceptance of the final product. Another benefit of early user involvement is reducing development costs and time by incorporating the identified requirements into the solution at an early stage of the development (Martin et al., 2012) and avoiding costly features that the users do not want or cannot use (Kujala, 2003). The involvement of different stakeholders helps developers to understand various contexts and possible limitations of the system’s usage. For example, when developing an ICT-based solution aimed at older adults, the developers must consider the particular characteristics of the target group such as their lack of experience in using digital solutions, lack of digital skills, lack of trust toward digital solutions, and physiological challenges they experience while using ICT-based solutions, such as poor eyesight, possible disabilities, and loss of hearing (Moore et al., 2010; Marston et al., 2019; Hirvonen et al., 2020). Peek et al. (2019) have pointed out that technological solutions aimed at supporting aging in place should be able to adapt to changes undergone by older, be robust in the sense that they can be used effectively when changes in abilities occur, and be capable of mitigating unfavorable changes. Failing to meet the needs of older adults can result in inefficient or even useless systems as they are not adopted by the target group. Some experience has been shared in scientific literature where the actual need was markedly different from the one envisaged by the developers (Martin et al., 2012). Therefore, the involvement of all of the stakeholders in the requirements elicitation phase is the key issue in user-centered approaches to ensure the usability and acceptance of the solution. However, it has been pointed out that despite the expanding field of ICT-based solutions aimed at older adults, there is a lack of knowledge about the needs and expectations of older adults and other stakeholders (Koch and Hägglund, 2009).

There are a variety of methods that can be used for requirements elicitation (Kujala, 2003). In selecting an appropriate method, it is important that it must be understandable to all participants—both engineers and non-technical stakeholders—such as older adults and caregivers without a technical background. Workshops, interviews, and focus groups are widely used for requirements elicitation (Martin et al., 2012; Van Velsen et al., 2013; Miller et al., 2015; Lorca et al., 2018; Taveter et al., 2019). Several researchers have highlighted the need for further research focusing on requirements elicitation processes, which would provide guidance for applying different elicitation methods (Koch and Hägglund, 2009; Martin et al., 2012; Van Velsen et al., 2013; Bjering et al., 2014). Today, in the situation where face-to-face meetings are not recommended or are even prohibited in several countries struggling with the outbreak of COVID-19, there is even a greater need for research and sharing of experience in methods of requirements elicitation and involvement of stakeholders.

Against the background described above, the aim of the current article is to share a unique experience from the Pharaon project where due to the COVID-19 outbreak alternative requirements elicitation methods and online tools were applied instead of face-to-face co-creation workshops. This situation provided us with a unique opportunity to seek answer to the following research question (RQ):

RQ1. How can face-to-face co-design workshops be replaced with alternative methods for requirements elicitation?

Previous literature has pointed out that there is a lack of knowledge about the needs and expectations of older adults and other stakeholders (Koch and Hägglund, 2009). In the current study, the involvement of the pilots from five European countries in the requirements elicitation process provided us a unique opportunity to address this gap and seek an answer also to the second research question:

RQ2. Which are the functional, quality, and emotional requirements common to different European countries aimed at supporting smart, healthy, and active living of older adults?

## Materials and Methods

### Pharaon Project

In December 2019, 41 partners and six pilots from 12 European countries joined their efforts in the Pharaon project (EU Grant Agreement 857188) to make smart and active living environments for the aging population of Europe a reality by creating a set of integrated, highly customizable, and interoperable open platforms with advanced services, devices, and tools. A user-centric approach is used in the Pharaon project to maximize the final usability and acceptance of the open sociotechnical ecosystem being created by all stakeholders. The main end users of the Pharaon ecosystem will be older adults, whereas several other stakeholders, such as healthcare professionals, formal and informal caregivers, volunteers, governmental and non-governmental organizations, and others, have been identified and involved. The input gathered during the requirements elicitation process was utilized for representing user and pilot requirements for all six pilot sites from five countries involved in the project. The requirements served as a foundation to define the initial architecture of the Pharaon sociotechnical ecosystem and will guide the project activities to achieve and deploy a successful final system. The six pilot sites involved in the project are: Italy, Netherlands, Andalusia in Spain, Murcia in Spain, Portugal, and Slovenia.

**Figure 1:**
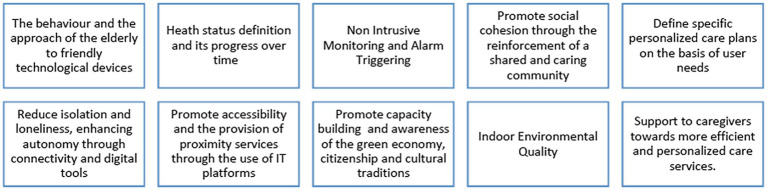
Challenges addressed by the Pharaon project.

The most relevant challenges defined at the beginning of the project that will be addressed with the ecosystem are presented in Figure 1.

### Requirements Elicitation

**Figure 2:**
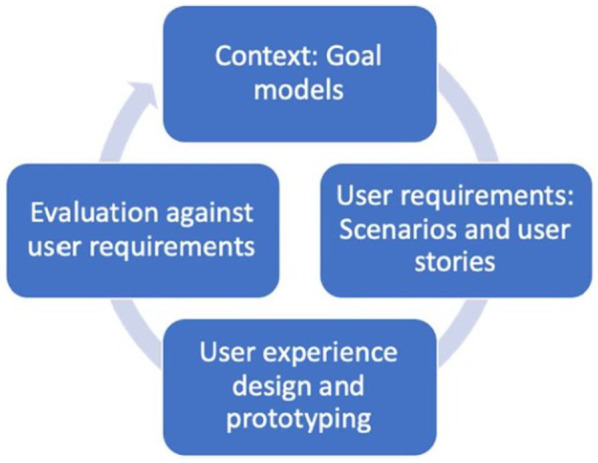
Framework for iterative co-design of the Pharaon ecosystem.

The requirements elicitation process of the project followed roughly the co-design and requirements elicitation process prescribed by the ISO 9241-210 standard (2019), which provides a framework for human-centered design activities and consists of four stages that are performed in several iterations: context of use, specification of user and organizational requirements, design solutions, and evaluation against requirements. The framework for iterative co-design of the Pharaon ecosystem is depicted in Figure 2.

The initial plan was to elicit requirements from stakeholders, particularly from older adults, in each pilot site through co-design workshops that would result in identifying functional, quality, and emotional goals proposed by the stakeholders. Several up-to-date methods of co-design for the elicitation of user requirements, such as DO-BE-FEEL™ (Lorca et al., 2018), HOW-NOW-WOW (Ericson and Törlind, 2013), and empathy maps (Ferreira et al., 2015), were planned to be used. It was agreed among the project partners that the requirements will be represented in terms of goals because goal-oriented requirements engineering is one of the state-of-the-art approaches in requirements engineering (Sterling and Taveter, 2009; van Lamsweerde, 2009; Horkoff and Yu, 2016). From among the approaches of goal-oriented requirements engineering, agent-oriented goal modeling and scenario modeling were chosen because they enable holistic representation of functional, quality, and emotional requirements (Sterling and Taveter, 2009; Miller et al., 2014, 2015; Lorca et al., 2018). In particular, motivational goal modeling explicitly addresses emotional requirements, rooted in the theory of constructed emotion (Taveter and Iqbal, 2021), which is of utmost importance for older adults (Miller et al., 2015; Taveter et al., 2019). Another advantage of motivational goal modelling is that it has been previously demonstrated to be a suitable and comfortable method for supporting the communication between technical and non-technical stakeholders (Sterling and Taveter, 2009; Shvartsman et al., 2010; Shvartsman and Taveter, 2011; Miller et al., 2015; Taveter et al., 2019; Mooses and Taveter, 2021). Additionally, the elicited goals can be easily turned into more concrete requirements in the form of user stories (Tenso et al., 2017) or prioritized lists of requirements (D’Onofrio et al., 2019; Fiorini et al., 2021).

Due to the COVID-19 outbreak and the lockdown in European countries, face-to-face co-creation seminars were impossible to carry out and contingency plans were developed for the involvement of stakeholders. Each pilot site chose alternative requirements elicitation methods that were deemed most suitable in their respective situations. These amendments were coordinated with local ethical committees in all pilot sites. The aim of the requirement elicitation remained the same—to provide an understanding of what activities by older adults and other stakeholders should be supported by the overall sociotechnical system to be designed, and what are the quality and emotional aspects of the activities to be supported.

To answer RQ1 “How can face-to-face co-design workshops be replaced with alternative methods for requirements elicitation?,” we describe in section “Requirements Elicitation Methods Used” the alternative methods and accompanying digital tools that were used together with their strengths and weaknesses. Resulting from requirements elicitation, functional, quality, and emotional goals and the roles required for attaining these goals were identified. Each pilot site created goal models based on their input from elicitation. Despite the different challenges proposed at the beginning of the project by the pilot sites, several functional, quality, and emotional goals overlapped between the pilot sites. These goals answer RQ2 “Which are the functional, quality, and emotional requirements common to different European countries aimed at supporting smart, healthy and active living of older adults?.” The goals common to different European pilots and countries that were elicited are presented in section “Requirements Supporting Smart, Healthy and Active Living of Older Adults”.

## Results

### Requirements Elicitation Methods Used

A total of 473 subjects (95 older adults and 378 other stakeholders) were involved in the requirements elicitation process. With 11 older adults, a face-to-face workshop was conducted before the outbreak of COVID-19, while the other 84 older adults were involved during COVID-19 restrictions remotely through other means. Similarly, 128 stakeholders were included in the requirements elicitation process through remote interviews and seminars. Additionally, 250 stakeholders were involved *via* online questionnaires. The stakeholders consisted of formal and informal caregivers, healthcare professionals, service and technology providers, and representatives of the public sector and universities.

When choosing an alternative requirements elicitation method, pilots had to consider several different aspects, such as restrictions applied in their countries, the level of computer and Internet usage, and the need for approvals by ethical committees. As a result, the following two categories of methods can be distinguished that were used for identifying the needs and problems of older adults and other kinds of stakeholders: (1) methods without digital technologies and (2) methods with digital technologies. Table 1 provides an overview of the requirements elicitation methods that were used by the pilot sites in the Pharaon project. Half of the pilot sites combined different methods to involve older adults and other stakeholders during COVID-19 restrictions. With a few exceptions, older adults were involved through methods without digital technologies, such as phone interviews. Although other researchers have conducted co-design workshops with older adults by digital means (e.g., Pedell et al., 2021), the digital literacy of the older adults involved and their trust toward technology enabled to conduct such workshops only in one of the pilots. Additional methods that were used for mapping the needs of older adults were reviewing the literature and exploring findings from previous projects. With other kinds of stakeholders, digital technologies were more frequently used. In sections “Face-To-Face Co-creation Seminar” to “Technologies Used”, we will present a short overview of the requirements elicitation methods that were applied in the Pharaon project. This is followed by section “Positive Outcomes, Barriers Faced and Lessons Learned” where we analyze the strengths and weaknesses of the method utilized.

**Table 1:** Requirements elicitation methods used for older adults and other stakeholders by pilot sites.

Method	Italy	Netherlands	Portugal	Slovenia	Andalusia (Spain)	Murcia (Spain)
*(1) Methods without digital technologies*						
Face-to-face co-creation seminar^*^		OA				
Phone interviews	OA; S				OA; S	
Review of literature and previous projects			OA; S	OA; S		OA; S
*(2) Methods with digital technologies*						
Online interviews	OA; S					
Online questionnaire	S					OA; S
Virtual co-creation seminar	S	S	S			OA; S
Semi-virtual co-creation seminar			OA			

OA, Older adults; S, other stakeholders.

* Conducted before COVID-19 outbreak.

#### Face-to-Face Co-creation Seminar

A face-to-face co-creation seminar was conducted by the Dutch pilot immediately before the outbreak of COVID-19 with older adults aged between 64 and 88 years. At the start of the workshops, the participants were asked to describe their typical day related to the four categories—food, movement, social contacts, outside activities—which were based on the aims of the Pharaon project. Participants wrote the elements of their day on differently colored post-its, indicating the four categories, and attached them to a poster. After discussing the posters with the group, they received another poster and were asked to recreate the first exercise, this time including what they would like to change or do differently in their daily life. In the last exercise, the participants were asked to write a short fantasy story describing how they could bridge the gap between the 2 days that were described.

#### Phone Interviews

The main reason for conducting phone interviews instead of using video conference systems was the lack of accessibility and the level of computer skills of older adults. One limitation of phone interviews was that the pilot sites were unable to use illustrative materials, which had to be replaced with short stories. This underlined the need for storytelling skills by requirements engineers. Storytelling was particularly important because there was no social interaction between the participants that would have supported the co-creation of unique and common scenarios of applying various technologies. At the same time, the interviews were semi-structured, which made them flexible and dynamic, allowing to adapt the interview questions based on the knowledge of the person on the topic. A strength of using phone interviews was the possibility to acquire the views and problems of less digitally skilled older adults. Also, it cannot be underestimated that phone interviews provided older adults with opportunities for additional interactions during the period of social isolation.

#### Findings From Previous Projects and Literature

Due to the restricted access to older adults and overall social isolation, three out of six pilots focused their attention on identifying previously validated requirements from earlier projects and literature. A limhitation of this approach is that the end users and stakeholders are not directly involved at the initial requirements elicitation stage. However, as the results of applying this method indicated, distinguishing significant requirements identified by the previous research allows to form initial goal models, which can be validated and improved later together with the stakeholders. Indeed, as was the case with the pilot sites of Murcia in Spain and Portugal, this method was accompanied by other methods, where older adults and other kinds of stakeholders were directly involved at later stages. Therefore, the initial literature-based requirements elicitation provided the pilot site frameworks that improved the efficiency of the communication processes with the stakeholders.

#### Online Interviews

Online interviews were carried out by the Italian pilot with those stakeholders and older adults who were able to use video conferencing systems in compliance with the General Data Protection Regulation (GDPR) regulations. The advantage of online interviews over phone interviews was the possibility to use illustrative materials, such as videos or pictures, to introduce the topic and share video clips, which gave a more personalized touch to the communication. However, similarly to phone interviews, the component of co-creation with other participants was missing, which can be considered as a limitation of this approach.

#### Online Questionnaires

Using online questionnaires had several aims. For example, for Italian pilot online questionnaires served as a tool to evaluate the attitude toward technology by the seminar participants. This helped to focus and tailor the group discussion. Online questionnaires also facilitated interactivity during the seminars. In the Murcian pilot, an online questionnaire was used to obtain an overview of the needs, situation, and background of older adults and different stakeholders. In addition, online questionnaire helped to identify older adults, relatives, and healthcare professionals interested in participating in the forthcoming phases of the pilot, including virtual co-creation seminars. Therefore, online questionnaires should be considered as a supplementary rather than standalone tool for requirements elicitation.

#### Virtual Co-creation Seminar

Only one pilot site conducted a virtual co-creation seminar with older adults, whereas four pilots out of six applied this kind of seminar with other kinds of stakeholders—caregivers and stakeholders dealing with technologies. The methods that were used for conducting virtual co-creation seminars were the DO-BE-FEEL™ (Lorca et al., 2018) method and the HOW-NOW-WOW method (Ericson and Törlind, 2013).

In the workshops conducted by the DO-BE-FEEL™ method, after the introduction of the project and the aim, the participants were asked to think: (1) what should a solution that supports the active and healthy aging of older adults *do*? (2) how should it *be*, i.e., what quality characteristics should it have? and (3) how should it make them *feel*? (4) *who* should do it, i.e., what *roles* should be involved? The corresponding keywords elicited from the participants were represented as a table consisting of the respective four columns. After the workshop, the keywords in the columns were arranged into a goal tree consisting of functional, quality, and emotional goals, and roles.

In the workshop conducted by the HOW-NOW-WOW method, three categories of ideas were identified from the discussions among the participants and grouped into three quadrants as follows: (1) NOW: Normal ideas, easy to implement; (2) HOW: Original ideas, impossible to implement; (3) WOW: Original ideas, easy to implement. Following, the best ideas were identified by voting by the participants, who were then divided into three groups to write down the scenarios for the best ideas belonging to the respective three categories.

Across all the pilot sites, the virtual co-creation seminars created a collaborative environment and facilitated discussions among the participants. A disadvantage of this approach was that through digital tools, it was hard to capture the attention by the participants for a prolonged time. This deficiency could be mitigated to some extent by creating smaller discussion groups and including different interactive modalities, such as pop-up questions and brainstorming by means of digital solutions, which increased the involvement by the participants. At the same time, compared with face-to-face seminars, the seminar moderators noticed the reduction of social interaction among the participants. Additional shortcomings of virtual co-creation seminars were having no opportunity for small talk with the participants and not being able to get to know them better. However, it was pointed out that attending virtual seminars was logistically more convenient compared with face-to-face seminars as it helped to save time that would have been needed for travelling for some participants. This benefit was particularly emphasized by the stakeholders who are highly occupied, such as healthcare professionals.

#### Semi-Virtual Co-creation Seminar

The Slovenian pilot conducted a semi-virtual co-creation seminar where the co-creation seminar with older adults was carried out by the caregivers of the residential unit housing older adults. The caregivers conducting the seminar were previously instructed by the project team and were provided with scripts for the seminar. The scripts consisted of guiding questions that helped to identify the needs of the older adults. The seminar was supported by the teleconference connection with the project team who were participating mainly as observers. The strength of such an approach was that the participants already knew their usual caregivers which made it easier for older adults to share their experiences and thoughts. However, the predefined scripts and the lack of previous experience in conducting such seminars by the caregivers reduced the flexibility and effectiveness of the seminar, as the caregivers conducting the seminar were not making any deviations from the provided scripts.

#### Technologies Used

The technology that was most frequently used for establishing an online connection with interviewees or seminar participants was Zoom, but Skype, Adobe Connect, and Microsoft Teams were also utilized. The selection of a tool significantly depended on the digital skills of the interviewees or participants but also on what was considered secure by the institution where the tool was used. The virtual workshops were accompanied by different solutions, which helped to increase the interactions with and between the participants, increase the involvement through short surveys, and support co-creation through enabling brainstorming and discussions in smaller groups. For group discussions in smaller groups, the breakout rooms’ feature of Zoom, Adobe Connect, or Microsoft Teams was used. The opportunity to create a breakout room within the meeting appeared to be very convenient for the participants as well as for the organizers because no additional web links for group discussions were needed. Moreover, when the group discussion was finished, the participants automatically returned to the initial meeting room. For documenting the ideas from brainstorming sessions and discussions in a manner that would be visible and editable for all participants, the pilot sites used Google Presentation, Google Docs or MURAL. The Italian pilot also used Google Forms for conducting short surveys during the seminars.

#### Positive Outcomes, Barriers Faced, and Lessons Learned

The most important positive outcome of using alternative elicitation methods was the involvement of all planned stakeholders. In some cases, using methods without digital technologies were used, such as phone interviews, enabled to include older adults with lower levels of digital literacy, who may not have been included under different circumstances. In addition, incorporating requirements from similar projects and literature further broadened the knowledge about the needs of the target group and helped to form a more exhaustive list of requirements which were validated with the stakeholders at later stages of the process. The application of the digital tools enabled several knowledgeable and experienced healthcare professionals to attend the virtual seminars and participate in the requirements elicitation process as virtual participation is less time-consuming compared with attending physical seminars. Another positive outcome was that even in a virtual setting, it was possible to create a collaborative and inclusive environment and fulfil the purpose of co-creation seminars.

Despite several benefits of applying digital tools for requirements elicitation, which offered an opportunity to involve in the elicitation process older adults and stakeholders without direct contact, several difficulties also had to be overcome. The barriers most frequently reported by the pilot sites were technical problems, such as the overall connectivity, and audio and video problems which were reported by 75% of the pilot sites who applied digital technologies for requirements elicitation. Another frequent challenge faced by the pilot sites was the overall digital literacy of older adults and their lack of understanding of technical terms or functionalities. This occasionally created confusion and often caused misunderstanding of a question, which resulted in rephrasing the question using a simpler language. Therefore, some bias can be present in the answers by the older adults. For the same reasons stated above, the moderators of the virtual seminar conducted with older adults were not able to apply usual techniques for warm-up and holding the attention that they typically use in face-to-face seminars, and alternatives had to be identified.

Table 2 summarizes the main lessons learnt that should be considered when planning a requirements elicitation session during COVID-19 or similar restrictions when a personal contact is not possible.

**Table 2:** Lessons learned from requirements elicitation during COVID-19 restrictions.

Lessons learned	Explanation
Be flexible when choosing the digital tool for communication	When choosing the digital tool for contacting stakeholders, the preferences and skills of the target group have to be considered. The digital tool used should be the means rather than the goal in itself. Focus should be put on applying commonly used tools to reduce the possible barrier caused by the frustration with unfamiliar or complicated digital tools. A creative approach of increasing the interactivity by means of virtual tools is recommended. Detailed guidelines on how to join the meeting should be provided.
Agree on rules and etiquette of a virtual seminar	To ensure the efficiency of virtual seminars, common rules and etiquette should be agreed or made available for the participants at the beginning of the seminar (e.g., switching off the microphone if not speaking, usage of the chat box, interruption of the speaker, etc.). This is especially important when participants have little experience in virtual seminars.
Provide enough time	It must be considered that setting up and/or joining a virtual seminar can take time and might need additional technical support by the organizers of the seminar. Choosing commonly used tools for interaction has the potential to reduce the risk of being unable to join the seminar. When the seminar is held with older adults, it is necessary to provide them with enough time for storytelling as their stories often help to reveal their actual needs and problems which is a valuable input for forming requirements of the ICT-based solution.
Use a variety of virtual tools to foster involvement	It is a challenge to retain the attention of the participants and create a co-creative environment in a virtual space. One way to achieve this is to make use of different virtual tools and features of communication tools, such as pop-up questions or shared documents where thoughts and ideas can be added online to make the seminar more interactive and fun. Also, keep an eye on the chat box to react on time to questions or comments by the participants.
Prefer smaller groups for discussions	When a seminar includes discussions, smaller groups should be created so that everyone would be able to express their thoughts, ideas and feelings. Special attention should be paid on this aspect during virtual seminars as people are not so prone to talk through digital tools.
Favor the use of video camera	Keeping the camera on supports the communication and involvement of participants. Moreover, it helps to refrain them from being involved in other activities, while they should be focusing on the seminar topic. Naturally, the connectivity and the privacy issues must also be considered here.
Make use of previous work and experience	Mapping the requirements from previous projects and research complements the information elicited from the stakeholders. Moreover, identifying some requirements beforehand can foster the discussions with the stakeholders and help to save their valuable time.

### Requirements Supporting Smart, Healthy, and Active Living of Older Adults

**Figure 3:**
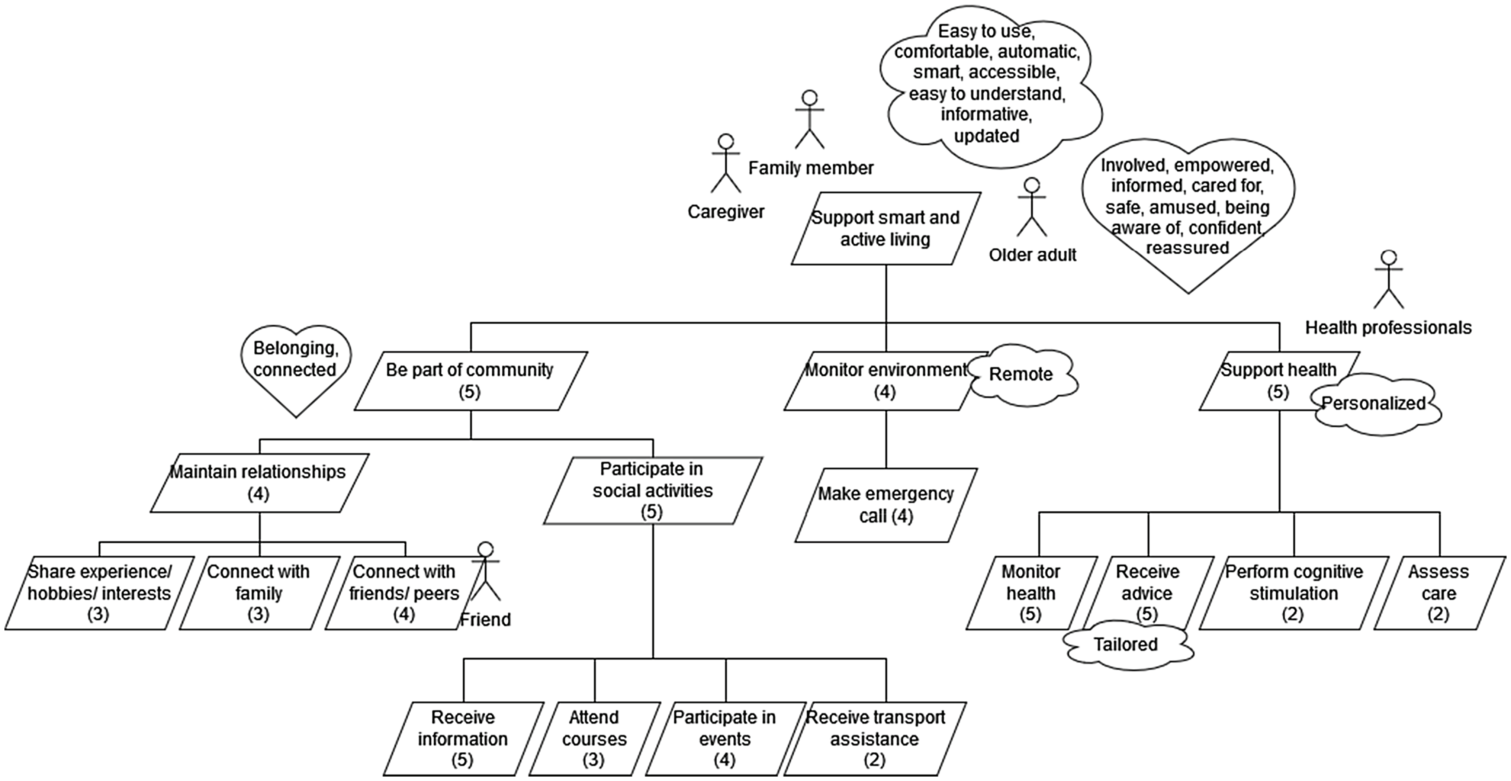
Similar functional, quality, and emotional goals identified. For each functional goal, the number of its occurrence is presented in the parentheses.

The requirements were elicited by the pilots of the Pharaon project using different methods overviewed in section “Requirements Elicitation Methods Used” and were then presented as agreed in the project in uniform way in the form of goal models (Sterling and Taveter, 2009; Miller et al., 2014, 2015; Lorca et al., 2018). Despite the fact that each pilot site had their own main challenges related to the ecosystem that is being created in the Pharaon project, a number of similar functional, quality, and emotional goals were identified during the requirements elicitation process. To provide a comprehensible overview of the identified common goals, the goals that emerged in most pilot sites were combined into one summary goal model that is presented in Figure 3. In the goal model, the functional goals are rendered in a tree-like hierarchy, where each sub-goal represents a particular aspect of achieving its parent goal. The functional goals are presented with tilted rectangles, while roles, quality, and emotional goals are attached to the appropriate functional goals and are, respectively, represented with stick man icons, and cloud and heart symbols.

The most prevalent functional goals were “Monitor health,” “Receive advice,” and “Receive information about activities/courses/initiatives” which were identified by 83% of the pilots which are sub-goals for “Support health” and “Participate in social activities” (Figure 3). The most prevalent quality goals reported by the pilot sites were “easy to use/comfortable” (100%), “personalized/tailored” (100%), “automatic/smart” (83%), “accessible” (50%), “easy to understand” (50%), “informative/guiding” (50%), “remote” (50%) and “updated” (50%). The most popular emotional goals were “involved” (100%), “empowered” (100%), “informed” (83%), “belonging/cared for” (83%), “safe” (67%), “amused/enjoying” (50%), “connected” (50%), “being aware of” (50%), “confident” (50%), and “reassured” (50%).

The most remarkable idiosyncratic functional goals elicited by the pilot sites were on the one hand the goals related to the natural environment by the Portuguese pilot, such as “raise awareness of green and blue areas” and “report needed environmental measures” and on the other hand the overly health-related goals identified by the Murcian pilot site, which are concerned with treatments, therapy schemes, and follow-up measures.

At the beginning of the project, all pilot sites identified the challenges of the Pharaon project applying to them (Figure 1). As a result of the elicitation process, several pilot sites identified additional challenges applying to them. The initial challenges together with the additional challenges that were identified by several Pharaon pilot sites during the requirements elicitation process are presented in Table 3.

**Table 3:** Challenges of the ecosystem addressed by the pilot sites.

Challenge	Italy	Netherlands	Portugal	Slovenia	Andalusia (Spain)	Murcia (Spain)
PCH1—The behavior and the approach of elderly to friendly technological devices	o	+	+		+	+
PCH2—Heath status definition and its progress over time	o	o	+	o	+	o
PCH3—Non-Intrusive Monitoring and Alarm Triggering	o	o	+	o		o
PCH4—Promote social cohesion	o	o	o	o	o	
PCH5—Define specific personalized care plan on the basis of user’s needs	o		+	o		o
PCH6—Reduce isolation and loneliness, enhancing the autonomy through connectivity and digital tools		o	o	o	o	
PCH7—Promote accessibility and the provision of proximity services through the use of IT platforms		+	o	+	+	+
PCH8—Promote capacity building and awareness on green economy, citizenship and cultural traditions			o			
PCH9—Indoor Environmental Quality				o		+
PCH10—Support to caregivers toward more efficient and personalized care services	o		+	o		+

o, initially proposed challenges; +, additionally identified challenges.

## Discussion

The results of this paper extend the current research literature in two ways. First, they provide a detailed information about different requirements elicitation methods and their combinations that could be applied during COVID-19 or similar restrictions where direct contact with stakeholders is impossible. The results determine on an unprecedented scale how face-to-face co-design methods can be replaced with alternative methods for requirements elicitation and what is the possible effect of applying a variety of alternative methods on the outcomes of the elicitation process (RQ1). Second, the analysis of the requirements elicited from six pilot sites originating from five European countries extends our knowledge and the current literature on functional, quality, and emotional requirements for ICT-based systems that support smart, healthy, and active living of older adults in Europe (RQ2). Following, in sections “Requirements Elicitation From Older Adults” to “Requirements Elicitation—Virtual Co-creation Seminars” we discuss in more detail some aspects of answering the research question RQ1. Finally, in section “Requirements for Supporting Smart, Healthy, and Active Living of Older Adults” we elaborate on answering the research question RQ2.

### Requirements Elicitation From Older Adults

Requirements elicitation has a major impact on the effectiveness and usability of ICT-based solutions that should satisfy the needs of end users (Kujala, 2003). Therefore, it is crucial to involve the stakeholders in the requirements elicitation process and to identify their problems and needs in an early stage of the development process. According to the review by Abelein and Paech (2015), user participation and involvement have a positive effect on user satisfaction and system usage. Van Velsen et al. (2013) and Bjering et al. (2014) have pointed out the need for guidance and experience sharing on how to include older adults in the co-creation process. Such need is extremely relevant in the current situation where COVID-19 still sets restrictions on applying commonly used requirements elicitation methods with direct contact, such as face-to-face workshops, interviews, and focus groups (Sterling and Taveter, 2009; Martin et al., 2012; Van Velsen et al., 2013; Miller et al., 2015; Lorca et al., 2018; Taveter et al., 2019). According to our experience gained so far from the Pharaon project, the major barrier to involving older adults in virtual requirements elicitation sessions is their lack of digital literacy which makes it difficult to use digital communication tools. The lack of digital literacy has also been previously identified as one of the challenges to using digital solutions by older adults (Moore et al., 2010; Hirvonen et al., 2020). Considering this challenge, for a considerable number of older adults, telephone interviews were used for requirements elicitation in the Pharaon project. For those older adults who were willing to use digital communication tools, it was extremely important to choose a tool which the older adults were familiar with and modify the elicitation process accordingly. This significantly helped to reduce negative emotions and frustration that can be caused by setting up a new digital communication channel and environment. According to our experience acquired from the Pharaon project, there is a need for a simple virtual technological solution that requires a minimal setup effort. This finding is also confirmed by a review by Rubinger et al. (2020).

Another challenge to the requirements elicitation was the overall knowledge of technical terms by older adults which made it difficult to discuss some aspects of the requirements especially when no virtual tool was available for providing visual explanations. This resulted in simplifications in the explanations, which may have caused some biases. Our experience with requirements elicitation from older adults also highlights the importance of reserving time for storytelling as the stories shared by older adults involve the needs and problems faced by them, which can be turned into requirements for the ICT-based system.

Phone and virtual interviews allowed for the involvement of the older adults in the elicitation process, although some limitations compared with face-to-face seminars were present. These limitations are in line with the previous research where the impossibility of using body language and technical issues has been reported (Peisachovich et al., 2020). However, our experience from the Pharaon project confirms that it is highly important to remain flexible in choosing the communication tool, because in the current study the willingness to use non-digital communication channels like phone enabled to involve in the elicitation process less digitally skilled older adults which provided invaluable input to the whole process of requirements elicitation.

In the Pharaon project, only the Murcian pilot site managed to conduct a completely virtual workshop with older adults (Martínez et al., 2021). Therefore, more experience sharing is needed on the guidance of involving older adults in virtual co-creation workshops. This furthermore points to the above-mentioned need for a simple virtual technological solution that requires a minimal setup effort. Despite the limitations of the alternative methods used for requirements elicitation, the methods that were used in the Pharaon project generally fulfilled the overall aim of the elicitation process. However, the validity of these methods will be re-evaluated at the later stages of the Pharaon project where the requirements are mapped to the architecture and building blocks of the Pharaon ecosystem.

### Requirements Elicitation—Findings From Previous Project and Literature

Another alternative method that was used for requirements elicitation in the Pharaon project, which seems to be underused in the requirements elicitation, is the review of literature and similar previous projects to identify possible functional, quality, and emotional requirements. Literature review is very common in research communities when, for example, an intervention is being planned (Mooses et al., 2021) or a research model is being developed (Zimmerman et al., 2007). In the research literature, the requirements elicitation process is often insufficiently described, and to the best of our knowledge, only a few studies have used findings from literature or previous projects in the context of requirements elicitation (Latulippe et al., 2019; Mooses and Taveter, 2021). This method complements other requirements elicitation methods. For example, it is possible that the participants of a co-creation workshop miss some important requirements as they focus on other requirements. To avoid this, integrating requirements from the literature or previous projects creates a more comprehensive set of requirements that can be validated with the stakeholders. Therefore, our experience is in line with Latulippe et al. (2019) who claim that building requirements on the requirements identified from previous projects and studies supplement the requirements proposed by the stakeholders and help to deepen the reflection. Equally important is the fact that involving literature reviews in the requirements elicitation process increases the evidence-based component of the ICT-based solution and helps to include the functionalities that have a potential to increase the usability and effectiveness of the solution.

At the same time, considering previous experience helps to avoid possible negative consequences on the end users (Hirvonen et al., 2020). For example, if we know that an issue such as infringement of privacy, distrust in data or information, or the effort needed to use the system has had negative impact on using an eHealth service by older adults (Hirvonen et al., 2020), we can pay extra attention to this aspect already in the requirements elicitation. Therefore, the analysis of previous projects and scientific literature have a great potential in supplementing the requirements elicitation process with invaluable input and increasing the effectiveness of the resulting ICT-based solution.

### Requirements Elicitation—Virtual Co-creation Seminars

In the Pharaon project, virtual co-creation seminars were mainly used for eliciting requirements from other kinds of stakeholders, such as formal and informal caregivers, healthcare professionals, and service providers. Based on the experience gained from the Pharaon pilot sites, such virtual co-creation seminars constitute a viable alternative to face-to-face seminars because the participants were actively involved and contributing. However, additional effort had to be exerted on incorporating a variety of virtual tools that foster the involvement and keep the participants focused on the topic. In our case, virtual tools that enable pop-up questions or enable simultaneous mind mapping and document editing features proved to be efficient for facilitating the involvement. In addition, our experience confirmed that group discussions should be carried out in smaller groups so that everyone would have an opportunity to express their opinion. A significant advantage of the virtual co-creation seminar highlighted by the participants was the time-saving aspect as there was no need to travel, and therefore, a larger number of healthcare professionals were able to join.

Overall, our experience gained in the Pharaon project indicated that combining different requirements elicitation methods, with or without using digital solutions, allowed for effective involvement of stakeholders despite their age or profession. Moreover, the inclusion of the literature reviews and results from previous projects extends the knowledge obtained from co-creation seminars and this way increases the evidence-based features of the resulting ICT-based solution.

### Requirements for Supporting Smart, Healthy, and Active Living of Older Adults

#### Functional Goals

Previously, the lack of knowledge about the needs and expectations of older adults and other stakeholders has been pointed out (Koch and Hägglund, 2009). Against this background, the Pharaon project offered a unique opportunity to address this gap by identifying common functional, quality, and emotional requirements for supporting smart, healthy, and active living of older adults in five European countries. This research result is summarized by the goal model depicted in Figure 3. The most prevalent functional goals that were common among the pilot sites of Pharaon were associated with health management (“Monitor health,” “Receive tailored advice”) and social interaction (“Receive information about activities/courses/initiatives,” “Socialize with friends and family”). This is well aligned with the results from previous research (Hirvonen et al., 2020), which indicate that eHealth services are predominantly used for health management and social engagement, such as communication, sharing information, and receiving feedback. In other words, the health management and social interaction features of ICT-based systems have a great significance for retaining mental and physical health and supporting aging in place.

Information and communication technology-based solutions for health management keep track of different health indicators, support healthy and active aging, provide suggestions, and track activities (Hirvonen et al., 2020). A promising approach for older adults seems to be smart home applications where the technological equipment that is used for monitoring of residents is integrated into the infrastructure of the residence (Demiris and Hensel, 2008; Moraitou et al., 2017). Solutions of this kind promote independence of older adults. They also require minimal training or operation, which makes them convenient and easy to use by older adults. This can be considered as an important strength of such smart home solutions because non-familiarity, lack of prior experience, usability issues, and fear of making a mistake have been previously identified as barriers to using ICT-based solutions by older adults (Hirvonen et al., 2020). At the same time, smart home solutions for older adults offer a sense of safety and security for the older people and their relatives as immediate actions can be taken in critical circumstances, such as falls or abnormal physiological signs. However, since another barrier to using technological solutions by older adults is the fear of losing privacy (Yusif et al., 2016; Marston et al., 2019; Hirvonen et al., 2020), special attention should be paid on ensuring privacy, confidentiality, and security of the collected data and raising the awareness of older adults about the privacy of the system.

Supporting social interaction seems to be obligatory for ICT-based home care systems aimed at older adults as they can have a significant effect on the physical and mental health of older adults (Cornwell and Waite, 2009; Santini et al., 2020). In fact, it has been shown that adequate social relationships decrease the mortality risk independently of the health status and cause of death (Holt-Lunstad et al., 2010). Besides reducing social isolation, enabling communication between older adults and caregivers or healthcare professionals improves the management of symptoms of diseases or conditions in daily life (Lindberg et al., 2013). This further emphasizes the importance of social interactions with peers, family members and healthcare professionals.

#### Quality and Emotional Goals

Quality goals that were most frequently reported by pilot sites were concerned with user experience—“simple to use” and “accessible.” Quality goals and emotional goals common to the pilot sites are also represented in Figure 3, just like the functional goals. Simplicity and ease of use have been highlighted as important features by older adults also in other studies (Heart and Kalderon, 2013; Yusif et al., 2016; Hirvonen et al., 2020). Moreover, providing tailored training and support during the implementation phase helps to get the older adults familiar with the ICT-based system and creates a positive attitude toward the system (González et al., 2015). Tailored training and support help to overcome the fears of older adults deriving from the lack of computer skills and experience, which has been identified as one of the barriers to using ICT-based solutions (Yusif et al., 2016; Hirvonen et al., 2020). Moreover, learning new ICT skills promotes self-confidence and self-esteem by older adults (González et al., 2015). During the training, it is important to teach new skills to older adults and also highlight the benefits, and point out relevant content and functionalities, which help to reduce the fears and increase the adoption of the solution (Heart and Kalderon, 2013; Hirvonen et al., 2020). Providing supportive training has the potential to increase the positive attitude toward the ICT-based solution and increase its usability (González et al., 2015). Considering that the lack of digital literacy and computer skills of older adults posed a challenge for the researchers in the Pharaon project during the requirements elicitation process, our experience confirms the need to provide the older adults with additional support and training during the solution adoption and implementation phase.

For the quality goals, the requirements elicitation performed in the Pharaon project indicated that the stakeholders value tailored, personalized, and smart solutions. Personalization is an important quality goal as it improves the user adherence (Monteiro-Guerra et al., 2020) and help to practice personalized patient-centered treatment where the treatment, therapy schemes, and follow-up measures are adapted to the conditions of a particular patient (Fico et al., 2016).

The two most frequently reported emotional goals by all of the pilot sites were “involved” and “empowered.” Therefore, as is already pointed out in section “Functional Goals”, supporting social interactions seems to be an indispensable feature that helps to retain the quality of life of an older adult in a home setting. Other emotional goals proposed by several stakeholders were “sense of belonging,” “being involved,” and “reassured.” These emotional requirements indicate the need for smart solutions, which can take into account the particular needs of each end user and adequately react to the information gathered by the monitoring systems.

The identified goals sound generic, but they constituted an important starting point for identifying scenarios for achieving the goals (Sterling and Taveter, 2009) and detailed requirements. The requirements were prioritized and elaborated according to the principles laid out by Fiorini et al. (2021) and by Tenso et al. (2017).

### Limitations

We acknowledge that the main weakness of the current study is the paucity of requirements validation. As of now, the functional, quality, and emotional goals have been identified and presented in the form of goal models. The scenarios for achieving the goals (Sterling and Taveter, 2009) have been created and the scenarios have been elaborated into detailed requirements based on the principles laid out by Fiorini et al. (2021) and by Tenso et al. (2017). The requirements have also been translated to the architecture of the Pharaon ecosystem but have not been validated yet because the initial iteration of designing and implementing the Pharaon ecosystem is still under way.

This study focused on identification of the needs as the first part of the iterative user-centered co-design process conducted in the Pharaon project. The co-design process is presented in Figure 2. We believe that using alternative methods in the COVID-19 situation helped to meet the objective of identifying the needs and problems of older adults and other kinds of stakeholders and established a strong foundation for the further software development and integration processes to be conducted in the Pharaon project. Another strength of the requirements elicitation process performed by us is that different kinds of stakeholders were involved, which helps to obtain a broader understanding of their needs and problems and helps to meet the scientific criteria of credibility. As the whole development process is planned to be agile and iterative, the functionalities identified during the requirements elicitation process will be amended based on the feedback by the stakeholders and older adults during the later development, piloting, and validation phases.

The extreme situation associated with the COVID-19 outbreak and lockdowns provided the project partners with an invaluable experience of requirements elicitation using alternative methods and incorporating online tools. We hope that our article inspires and supports the involvement of end users in the requirements elicitation process to provide solutions that meet the needs of end users even under such challenging circumstances. We encourage other researchers to share their experience with requirements elicitation by means of digital solutions during the COVID-19 restrictions to provide guidance to the research community.

## Conclusion

Our experience with requirements elicitation under the circumstances created by the spread of COVID-19 confirmed that there are acceptable alternative methods to face-to-face co-creation seminars that effectively involve older adults and other stakeholders in the elicitation process. As for the alternative methods without virtual components, the lack of co-creation elements must be acknowledged. At the same time, some methods, such as the review of previous literature and projects, have a potential to strengthen the requirements elicitation process through creating a broader context for the ICT-based solution to be designed and involving evidence-based components. As for the alternative methods with a virtual component, even if human contact is essential, virtual co-creation seminars can be considered as an acceptable method for active involvement of stakeholders in the co-design process. We showed that despite the requirements elicitation method used, the results can be effectively presented in the form of a goal model that facilitates the communication between technical and non-technical stakeholders. Even more importantly, we identified common functional, quality, and emotional requirements for supporting smart, healthy, and active living of older adults in five European countries and summarized this research result by the overall goal model of the Pharaon project.

## Data Availability Statement

The original contributions presented in the study are included in the article/supplementary material, further inquiries can be directed to the corresponding author.

## Ethics Statement

Ethical review and approval was not required for the study on human participants in accordance with the local legislation and institutional requirements. Written informed consent for participation was not required for this study in accordance with the national legislation and the institutional requirements.

## Author Contributions

KM wrote the first draft of the manuscript. The design of requirements elicitation was led by KT with in close collaboration with other authors. All authors contributed to the collection of data, manuscript revision, read, and approved the submitted version.

## Funding

The research work reported in this article has received funding from the Pilots for Healthy and Active Ageing (Pharaon) project of the European Union’s Horizon 2020 research and innovation programme under the grant agreement no. 857188 and from the European Social Fund *via* the IT Academy programme. This article is based upon work from COST Action CA16226 SHELD-ON—Indoor living space improvement: Smart Habitat for the Elderly, supported by COST (European Cooperation in Science and Technology). COST (European Cooperation in Science and Technology) is a funding agency for research and innovation networks. Our Actions help connect research initiatives across Europe and enable scientists to grow their ideas by sharing them with their peers. This boosts their research, career and innovation. More information in www.cost.eu.

## Conflict of Interest

The authors declare that the research was conducted in the absence of any commercial or financial relationships that could be construed as a potential conflict of interest.

## Publisher’s Note

All claims expressed in this article are solely those of the authors and do not necessarily represent those of their affiliated organizations, or those of the publisher, the editors and the reviewers. Any product that may be evaluated in this article, or claim that may be made by its manufacturer, is not guaranteed or endorsed by the publisher.
